# Room-temperature ferroelectricity in MoTe_2_ down to the atomic monolayer limit

**DOI:** 10.1038/s41467-019-09669-x

**Published:** 2019-04-16

**Authors:** Shuoguo Yuan, Xin Luo, Hung Lit Chan, Chengcheng Xiao, Yawei Dai, Maohai Xie, Jianhua Hao

**Affiliations:** 1 Department of Applied Physics, The Hong Kong Polytechnic University, Kowloon, Hong Kong PR China; 2 School of Physics, Sun Yat-sen University, Guangzhou, 510275 PR China; 3 Department of Physics, The University of Hong Kong, Pokfulam Road, Hong Kong, PR China

## Abstract

Ferroelectrics allow for a wide range of intriguing applications. However, maintaining ferroelectricity has been hampered by intrinsic depolarization effects. Here, by combining first-principles calculations and experimental studies, we report on the discovery of robust room-temperature out-of-plane ferroelectricity which is realized in the thinnest monolayer MoTe_2_ with unexploited distorted 1T (*d*1T) phase. The origin of the ferroelectricity in *d*1T-MoTe_2_ results from the spontaneous symmetry breaking due to the relative atomic displacements of Mo atoms and Te atoms. Furthermore, a large ON/OFF resistance ratio is achieved in ferroelectric devices composed of MoTe_2_-based van der Waals heterostructure. Our work demonstrates that ferroelectricity can exist in two-dimensional layered material down to the atomic monolayer limit, which can result in new functionalities and achieve unexpected applications in atomic-scale electronic devices.

## Abstract (web summary)

It is difficult to maintain ferroelectricity in the two dimensional limit. Here, the authors report robust room-temperature ferroelectricity in the thinnest monolayer MoTe_2_ due to relative atomic displacements of Mo and Te atoms.

## Introduction

Ferroelectric materials have attracted intensive interest due to their broad applications in smart sensors, capacitors, transducers, actuators, energy harvesting devices, and non-volatile memories^1–6^. Ferroelectricity at the nanoscale has emerged as fertile ground for new physics and applications^7,8^. However, the depolarization field presents fundamental challenge for the ultrathin ferroelectric materials^9–12^. There is a critical thickness in the ferroelectrics, below which the ferroelectricity will disappear due to the increasing depolarization field in the thin films with decreasing thickness, as a result of the uncompensated charges at the interface. The critical thickness in the ferroelectric materials, such as typical prototype perovskite oxides ABO_3_, is limited by the scale of a few unit cells^10,11^. In addition, the Curie temperature (*T*_c_) for retaining ferroelectrics significantly decreases as the film becomes thinner. Recently, two-dimensional (2D) layered materials have attracted tremendous attention due to their novel appeal in both fundamental studies and potential applications^13–15^, presenting unprecedented possibilities to overcome the problems. It is noticeable that in-plane ferroelectricity is experimentally reported in SnTe thin-films with 1–4 unit cells^16,17^. For the SnTe with non-layered structure, however, the Curie temperature (*T*_c_) of the single-unit cell SnTe film is below room-temperature and in-plane ferroelectricity is not suitable for many promising applications. In addition, a few 2D materials are reported to exhibit few-layer room-temperature ferroelectricity, such as 4 nm CuInP_2_S_6_ (~6 layers)^18^, 3 nm In_2_Se_3_ (~3 layers)^19–23^, and 1.4 nm WTe_2_ (bilayer)^24^. Recently, there are several theoretical predictions on the ferroelectric phenomena in monolayer 2D materials, but still lacks the experimental observations on them^25–28^.

In this work, we report experimental observation of room-temperature ferroelectricity in an unexploited phase of MoTe_2_ down to the monolayer limit. Physical mechanism behind the phenomenon is understood by combining structural characterizations and theoretical calculations. Furthermore, we construct an ultra-thin van der Waals (vdW) heterostructure based ferroelectric tunneling junction (FTJ) by using the newly discovered monolayer ferroelectrics. Such a ferroelectric all-2D vdW heterostructure platform paves the way of developing thinnest ferroelectric devices, which will facilitate the miniaturization of next generation memory and atomic-scale logical devices.

## Results

### Phase transition

**Fig. 1:**
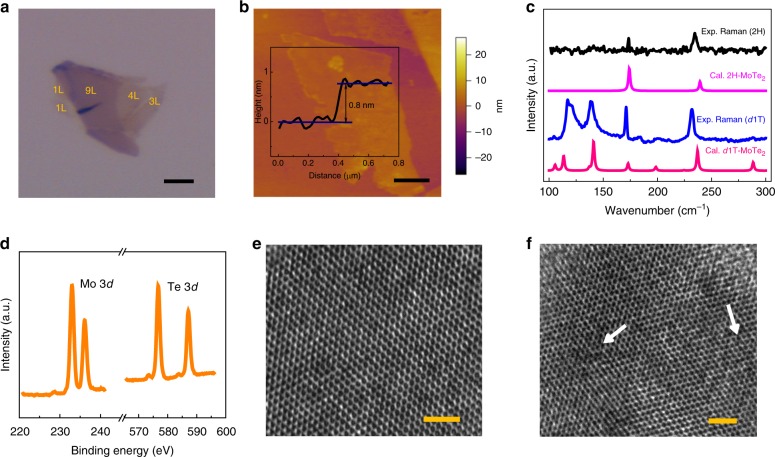
Phase transition of *d*1T-MoTe_2_. **a** OM image of *d*1T-MoTe_2_/Pt, scale-bar, 3 μm. **b** AFM image and corresponding height profile of monolayer *d*1T-MoTe_2_, scale-bar, 0.5 μm. **c** Experimental and calculated Raman spectra of 2H-MoTe_2_ and *d*1T-MoTe_2_. **d** XPS spectra of *d*1T-MoTe_2_. **e**, **f** HRTEM images of 2H and *d*1T-MoTe_2_ (arrows denote Te defects, scale-bar, 2 nm)

Ferroelectric MoTe_2_ nanosheets exfoliated on Pt substrate (Pt/Ti/SiO_2_/Si) were prepared by laser process^29^. Figure 1a, b shows optical microscopy (OM) and atomic force microscopy (AFM) images of MoTe_2_ nanosheets, respectively. The corresponding height profile of single-layer reveals a thickness of ~0.8 nm, as expected for monolayer MoTe_2_^30^. More images of the layers are seen in Supplementary Fig. 1a, b. Raman spectra of MoTe_2_ before and after laser processing are shown in Fig. 1c. The pristine 2H-MoTe_2_ nanosheets exhibit two main Raman modes, including A′_1_ mode close to 174 cm^−1^ and E′ mode close to 235 cm^−1^. Notably, a different set of Raman peaks was found in the new phase of MoTe_2_ after laser processing, referred to the distorted 1T (*d*1T) MoTe_2_. Based on the density functional perturbation theory described in Supplementary Note 1, we calculated the non-resonant Raman spectra of the monolayer 2H and *d*1T-MoTe_2_, which accord well with the experimental observations. The other Raman active modes with the corresponding irreducible representations are summarized in Supplementary Table 1. X-ray photoelectron spectroscopy (XPS) confirmed the elemental composition and chemical environment in the *d*1T-MoTe_2_ (Fig. 1d). The prominent Mo 3*d* and Te 3*d* peaks correspond to Mo–Te bonds. The phase transition involving defect behavior was further illustrated by high-resolution transmission electron microscopy (HRTEM). The 2H phase sample in Fig. 1e remains hexagonal symmetry without observable Te vacancies. However, some Te vacancies were found in *d*1T-MoTe_2_ (Fig. 1f), in agreement with the energy dispersive spectroscopy (EDS) results (Supplementary Fig. 1c, d). These results imply that the Te vacancies may be as a signature during the 2H-*d*1T phase transition^29^. Moreover, a similar 2H-*d*1T phase transition by laser irradiation was found in the MoTe_2_ exfoliated onto monolayer graphene (Supplementary Fig. 2).

### Ferroelectricity in *d*1T-MoTe_2_

**Fig. 2:**
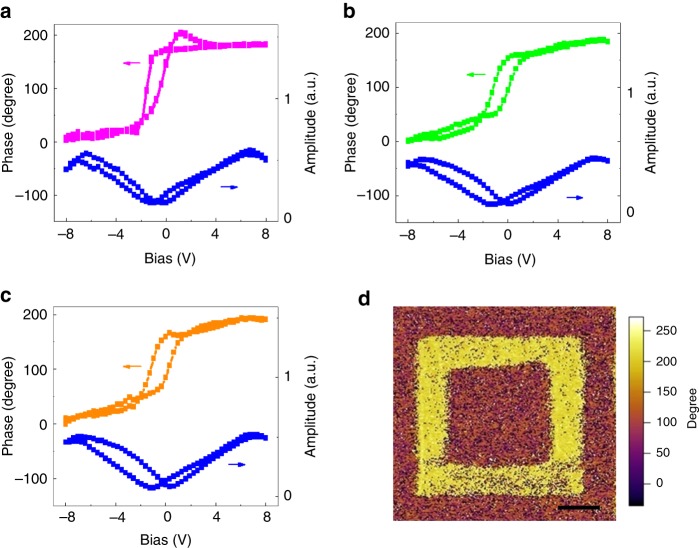
Ferroelectricity in *d*1T-MoTe_2_. **a** PFM phase hysteretic and butterfly loops of monolayer *d*1T-MoTe_2_. **b**, **c** PFM phase hysteretic and butterfly loops of *d*1T-MoTe_2_ before and after 30 days. **d** PFM phase image of monolayer *d*1T-MoTe_2_, where the electrical poling was applied by writing two square patterns with ±8 V, scale bar, 1 μm

Piezoresponse force microscopy (PFM) is a powerful tool to demonstrate the existence of a switchable ferroelectric polarization in ultrathin samples. Figure 2a reveals out-of-plane local PFM hysteretic loops in phase and amplitude recorded from monolayer *d*1T-MoTe_2_ at room temperature. The phase difference between the two polarization states is 180° and the minima in the amplitude loop coincides with the switching voltages in the phase signal, indicating a room-temperature ferroelectric nature. The existence of ferroelectricity is evident in MoTe_2_ nanosheets with different layer number (Supplementary Fig. 3a). To exclude the charging effects on the observed ferroelectricity, the PFM phase of few-layer MoTe_2_ was checked and there was little change in the ferroelectricity after 1 month (Fig. 2b, c). These results indicate that mono- or few-layer *d*1T-MoTe_2_ is essentially ferroelectrics with switchable spontaneous polarizations^21^. Similar results can be obtained in the *d*1T-MoTe_2_ on different substrates (Supplementary Fig. 3b). To study the potential ferroelectricity at a larger scale, we measured PFM phase image of *d*1T-MoTe_2_ (Fig. 2d). The PFM phase contrast reveals that the polarization is anti-parallel in the two domains, confirming the existence of ferroelectricity in the *d*1T-MoTe_2_. In comparison, to rule out the possibility with non-ferroelectric materials, the phase hysteretic loops measured at random locations on the surface of the 2H and *d*1T phase monolayer MoTe_2_. There is almost no phase contrast in pristine 2H-MoTe_2_, indicating the lack of ferrelectricity in 2H-MoTe_2_. On contrast, the *d*1T-MoTe_2_ shows the obvious ferroelectric hysteretic behavior (Supplementary Fig. 3c, d). We also compared the in-plane PFM signals of *d*1T-MoTe_2_ (Supplementary Fig. 3e, f), and the phase contrast is quite small, suggesting no obvious spontaneous polarization along in-plane direction. Intriguingly, the monolayer *d*1T-MoTe_2_ exhibiting room-temperature out-of-plane ferroelectricity is the thinnest compared to previously reported nanoscale ferroelectrics (Supplementary Table 2). It is known that the ferroelectricity in traditional oxide ultrathin films is affected by many factors, such as defects, surface reconstruction, and strains. In contrast, 2D layered structures ensure unique electronic properties since there are no constraints of lattice mismatch and incompatibilities of other materials. Therefore, the measurement of intrinsic ferroelectricity from 2D layer is not suffered from those effects. The availability of the 2D ferroelectric layer offers a rich playground for understanding intrinsic ferroelectric properties ultimately.

### Atomic structure and theoretical calculations

**Fig. 3:**
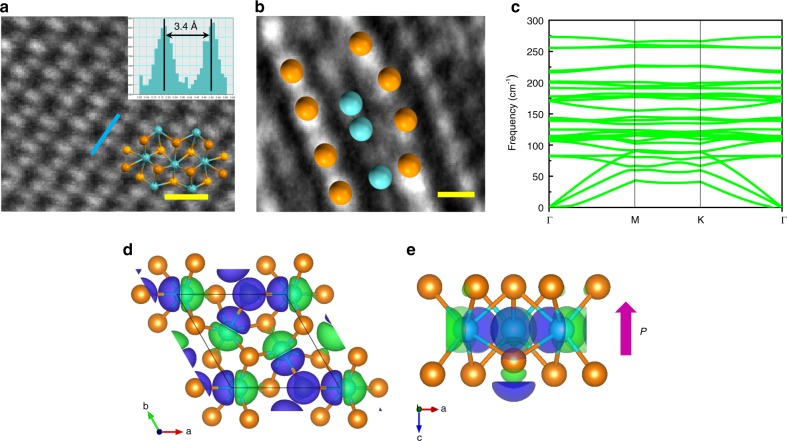
Atomic structure and theoretical calculations on ferroelectricity in *d*1T-MoTe_2_. **a** Top-view HRTEM image and intensity profile, the atomic structure of *d*1T-MoTe_2_ is placed on top, scale-bar, 0.5 nm. **b** Atomic structure image of monolayer *d*1T-MoTe_2_ and the inset shows atomic structure model (cyan and orange colors represent Mo and Te atoms, respectively), scale-bar, 2 Å. **c** Phonon dispersion of *d*1T-MoTe_2_. **d**, **e** Top-view and side-view of charge density difference between ferroelectric *d*1T and paraelectric 1T phases (green, purple, cyan, orange, and pink colors denote negative charge, positive charge, Mo atom, Te atom, and polarization, respectively)

In order to understand the ferroelectric mechanism in deep, we examined the atomic structure of *d*1T-MoTe_2_ as shown in Fig. 3a, b. For these measurements, the MBE grown samples under laser process were prepared^31^, and then similarly measured as above (Supplementary Fig. 4a–g). Such wafer-scale *d*1T-MoTe_2_ samples facilitate further characterizing the atomic structure by cross-sectional aberration-corrected scanning transmission electron microscopy (AC-STEM). According to HRTEM images, the distance between Te and Te atoms is 3.4 Å (Fig. 3a), exhibiting trimerized structure of *d*1T-MoTe_2_. The result is similar to the following calculation on non-centrosymmetric trimerized structure. The second harmonic generation signal can also be observed in monolayer *d*1T-MoTe_2_, further implying the non-centrosymmetric structure of *d*1T-MoTe_2_ (Supplementary Fig. 4h). In addition, the vertical atomic structure is evident from cross-sectional AC-STEM images, as revealed in Fig. 3b. It is found that a few Te atoms move towards the Mo plane in the out-of-plane direction by around 0.6 Å while the others largely remain still, which is consistent with the atomic structure model optimized from density functional theory (DFT) calculations, as discussed below. Such trimerized structure with vertical atomic displacement of Te causes spontaneous polarization.

To elucidate the underlying mechanism of ferroelectricity phenomena in the specific unexploited phase of MoTe_2_, we perform first-principles calculation to illustrate the *d*1T phase and investigate the microscopic origin of the ferroelectricity. The distortion associated with the K point instability leads to the trimerized$\sqrt 3 \times \sqrt 3$* d*1T-MoTe_2_ structure^25,28^. The thermodynamic stability of the *d*1T-MoTe_2_ can be confirmed by the fact that there is no imaginary frequencies existed in the phonon dispersion (Fig. 3c, Supplementary Fig. 5a, b). Previous studies suggest that the *d*1T-MoTe_2_ is an improper ferroelectric, where the spontaneous polarization occurs as secondary order parameters originated from the $\Gamma _2^ -$ distortion by translating Mo and Te atoms in opposite directions along the *c* axis^25,28^. Our in-depth DFT calculations suggest that the spontaneous polarization is mainly resulted from the process of the K_3_ trimerization with the polarization mainly arisen from the electronic part, while the $\Gamma _2^ -$ distortion has minor effect on the polarization (Supplementary Fig. 5c–f). From the difference of charge density between the *d*1T and 1T phase in Fig. 3d, e, the in-plane polarization is zero when summing all the dipoles together, which is in good agreement with aforementioned measurements. The net dipole only exists in the out-of-plane direction, resulting in the robust ferroelectricity perpendicular to the lattice plane as confirmed by the above experiments.

### FTJ devices

**Fig. 4:**
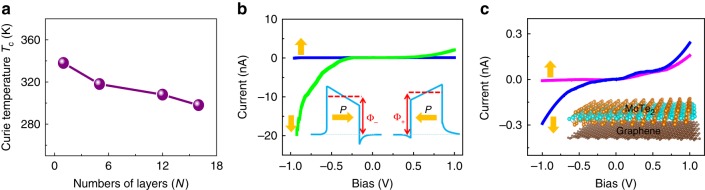
*T*_c_ of *d*1T-MoTe_2_ and FTJ devices. **a** Layer-dependent *T*_c_ of *d*1T-MoTe_2_. **b** *I*–*V* characteristic of monolayer *d*1T-MoTe_2_ on Pt and inset shows the energy diagram of FTJ devices. **c** *I*–*V* characteristic of few-layer *d*1T-MoTe_2_ on graphene and inset shows the schematic of device structure

In addition to the observation of ferroelectricity, *T*_c_ is of great importance to determine material’s usefulness in various applications. Figure 4a shows layer-dependent *T*_c_ in *d*1T-MoTe_2_, where *T*_c_ increases when decreasing layer number. Importantly, the *T*_c_ is above room temperature for monolayer and a few layers below around 16 layers (Supplementary Figs. 6 and 7). Owing to the chemically inert surface and weak vdW interlayer interaction, the 2D layer ferroelectric can be readily incorporated with counterparts (e.g., graphene and 2D semiconductors) to build functional heterostructure devices. Compared with the traditional ferroelectric random access memory, the FTJ-based memory has the advantage of non-destructive reading and electric field control of large resistance ratio. Especially, for the 2D vdW FTJ, the physical properties of the component layers are largely unaffected each other due to the weak vdW interaction, allowing for the design of heterojunction devices with desired functionality. Our monolayer *d*1T-MoTe_2_ ferroelectric has the merits of thinnest thickness, out-of-plane polarization and above room-temperature *T*_c_, thus it is suitable for constructing non-volatile FTJ devices at nanoscale^7,12,32,33^. Figure 4b shows the *I*–*V* curve of the FTJ measured by conductive atomic force microscopy (CAFM). The two polarization directions stand for the ON and OFF states of the device and the *I*–*V* curve is highly nonlinear in the ON and OFF states, and the ON/OFF resistance ratio of the FTJ device is about 1000. The observed *I*–*V* curves measured on different devices presented similar results, indicating good reproducibility. Moreover, 2D vdW *d*1T-MoTe_2_/graphene FTJ device is constructed and the measured *I*–*V* curve is presented in Fig. 4c. The vdW interaction has little effect on the intrinsic properties of the *d*1T-MoTe_2_ (Supplementary Fig. 8). Therefore, the vdW heterostructure can largely retain the intrinsic ferroelectric feature. The out-of-plane ferroelectricity existed in the *d*1T-MoTe_2_ exhibits great promising applications in modern electronic devices.

In summary, we report the observation of room-temperature out-of-plane ferroelectricity in the monolayer MoTe_2_ with unexploited *d*1T phase illustrated by our theoretical calculations. With the monolayer ferroelectric, ferroelectric device approaching to the ultimate limit is demonstrated. The discovery of ferroelectricity in monolayer 2D materials will facilitate the exploration of fundamental physics of ferroelectrics at the nanoscale, and opens up new possibilities for promising applications.

## Methods

### Preparation of materials

The MoTe_2_ nanosheets were prepared using mechanical exfoliation. The 2H phase MoTe_2_ nanosheets were processed by a laser with the power of 2–20 mW for 10–300 s under ambient condition. The laser wavelength is 488 nm and the areal power intensity is estimated as 2.5 mW/μm^2^. In addition, some MoTe_2_ samples were fabricated by molecular beam epitaxy (MBE). Monolayer MoTe_2_ films deposited on highly oriented pyrolytic graphite (HOPG) and SiC substrates from Mo and Te sources by a customized Omicron MBE reactor with a base pressure of 5 × 10^−10^ mbar. The fluxes of Mo and Te were generated from an e-beam and a conventional Knudsen cell, respectively. To ensure the stoichiometry and crystallinity of epitaxial MoTe_2_ film, the latter (Te) was set a few tens to hundred times that of Mo. The film growth rate was about 0.3 MLs h^−1^ by modulating the flux of Mo, which was determined by postgrowth measurement by scanning tunneling microscopy (STM). The growth temperature was ranged between 250 and 400 °C. Prior to MoTe_2_ deposition, the substrate was thoroughly degassed in the ultra-high vacuum chamber and flashed up to 600 °C.

### Characterization methods

The Raman spectra of samples were measured by Raman spectroscopy (Horiba, HR800) using a laser excitation source with the wavelength of 488 nm and the spot size of 1 μm. Microstructures and chemical compositions were examined by transmission electron microscope (TEM, JEOL JEM 2100F) equipped with energy-dispersive X-ray spectroscopy (EDX). The cross-sectional TEM specimen was prepared by applying FIB (JEOL JIB-4500) milling and lift-off technique. The cross-sectional atomic structure was performed in a JEOL JEM-ARM300F with STEM aberration corrector operated. The chemical composition of samples was measured using XPS (Thermol Escalab 250Xi, Al Kα radiation). A Ti:sapphire femtosecond laser with the wavelength of 990 nm was used to examine the second-harmonic generation (SHG) signals of samples. Morphological measurement of the samples was carried out using either a room-temperature Omicron STM system connected to the MBE chamber or a low-temperature Unisoku STM facility ex situ at 77 K. PFM measurements were carried out on a commercial atomic force microscope (Asylum Research MFP-3D), where a tip was driven with an ac voltage (*V*_ac_ = 0.5–2 V) under the tip-sample contact resonant frequency (~350 kHz). Hysteresis loops were collected in the DART (dual a.c. resonance tracking) mode. PFM images were taken in single frequency or dual frequency resonance tracking mode. In addition, vector PFM was also performed by imaging the in-plane signal. The *I*–*V* curves of the devices were measured by CAFM at room temperature to characterize the electrical transport characteristics.

### Calculation method

First-principles calculations of the electronic structure, phonon dispersion were performed within DFT as implemented in the plane-wave pseudopotential code VASP. The local density approximation (LDA) to the exchange-correlation functional was employed in the projected augmented wave throughout the calculation. Spin–orbit coupling effects were included in the calculations. We used the energy cutoffs of 450 eV for the truncation of the plane wave basis. Monkhorst–Pack k-point mesh of 15 × 15 × 1 and 7 × 7 × 1 was used to sample the Brillouin zones for 1T and *d*1T-MoTe_2_, respectively. The slabs were separated by 16 Å of vacuum to prevent interactions between slabs. All the atomic coordinates and lattice constants were optimized until the maximum component of the Hellmann–Feynman force acting on each ion was less than 0.003 eV/Å. The phonon dispersion was calculated using the finite displacement method in the 5 × 5 × 1 and 3 × 3 × 1 supercell for the 1T and *d*1T structures, respectively, where atoms were displaced to get the force constants that could build the dynamical matrix. The Raman frequencies and intensities of zone center phonons are calculated within density functional perturbation theory as implemented in the QUANTUM-ESPRESSO package. The DFT simulated Raman spectra are obtained with an artificial Lorentzian broadening based on the calculated non-resonant Raman intensity.

## Supplementary information

Supplementary Information

## Data Availability

The data are available from the corresponding authors upon reasonable request.
