# Supplementary material for: Room-temperature ferroelectricity in MoTe2 down to the atomic monolayer limit
Source: Nat Commun. 2019 Apr 16;10:1775. doi: 10.1038/s41467-019-09669-x (PMC6467908; doi:10.1038/s41467-019-09669-x)
Supplement: Supplementary file 1 — Supplementary Information [file 41467_2019_9669_MOESM1_ESM.pdf]

Supplementary Information

Room temperature ferroelectricity in $MoTe_2$ down to the atomic monolayer limit

Yuan *et al.*

Room temperature ferroelectricity in $MoTe_2$ down to the atomic monolayer limit

Shuoguo Yuan[1†], Xin Luo[1,3†], Hung Lit Chan[1], Chengcheng Xiao[1], Yawei Dai[2], Maohai Xie[2], Jianhua Hao[1]*

[1]Department of Applied Physics, The Hong Kong Polytechnic University, Kowloon, Hong Kong, P. R. China.

[2]Department of Physics, The University of Hong Kong, Pokfulam Road, Hong Kong, P. R. China.

[3]School of Physics, Sun Yat-sen University, Guangzhou, 510275, P. R. China.

[†]These authors contributed equally to this work.

* Corresponding author. Email: jh.hao@polyu.edu.hk

Supplementary Note 1. Structural characterizations of $MoTe_2$

The atomic force microscopy (AFM) image of $MoTe_2$ nanosheets with different thicknesses is shown in Supplementary Figure 1a. Supplementary Figure 1b shows cross-section TEM image of *d*1T-$MoTe_2$, indicating the layered structure, and the nanosheet thickness of each layer is about 0.8 nm, which is consistent with the AFM measurement. Supplementary Figure 1c,d gives energy dispersive spectroscopy (EDS) of 2H and *d*1T phase $MoTe_2$, the atomic ratio of 2H phase sample is around 1:2 and the atomic ratio of *d*1T phase sample is around 1:1.8.

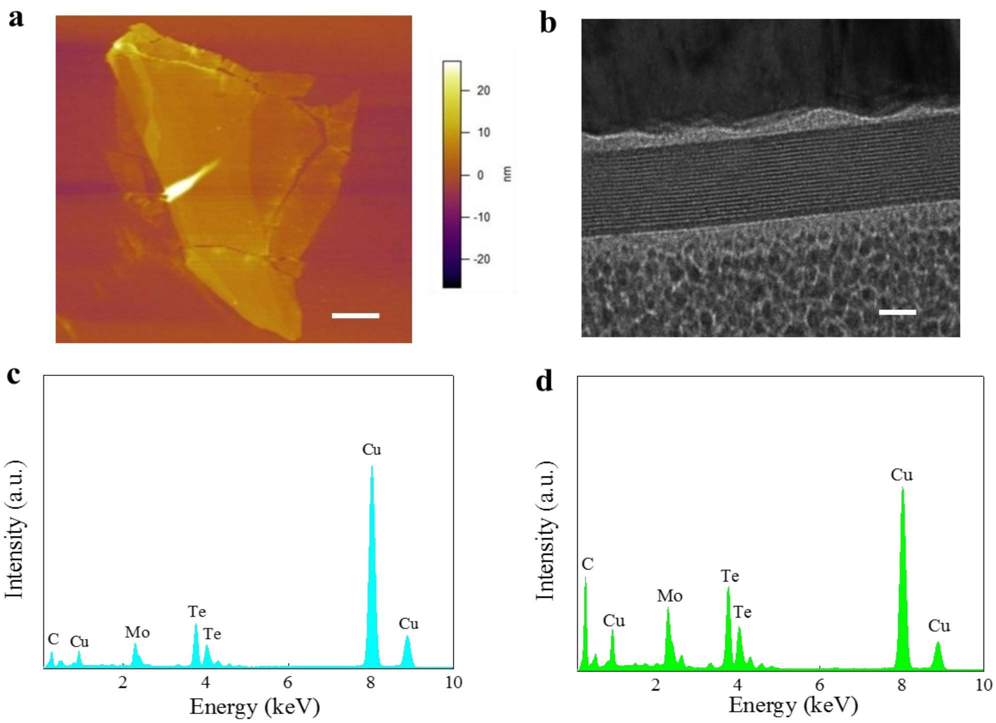

Supplementary Figure 1. Structural characterizations of $MoTe_2$. a, AFM image of *d*1T-$MoTe_2$ nanosheets onto Pt substrate (scale bar, 2 μm). **b**, Cross-sectional TEM image of *d*1T-$MoTe_2$ (scale bar, 6 nm). **c**,**d**, EDS curves of 2H-$MoTe_2$ and *d*1T-$MoTe_2$.

The good agreement between the calculated Raman spectra with the experiments in the 2H phase suggests that the DFT calculations can reproduce the Raman scattering spectroscopy for the $MoTe_2$ system. The characteristic Raman modes of *d*1T-$MoTe_2$ are consistent with the experimental observations (Supplementary Table 1). The seven typical modes (288 cm^{-1}, 237 cm^{-1}, 199 cm^{-1}, 173 cm^{-1}, 141 cm^{-1}, 114 cm^{-1} and 106 cm^{-1}) exhibiting larger Raman intensities are consistent with the experimental observations (experimental values are around 288 cm^{-1}, 232

cm^{-1}, 200 cm^{-1}, 171 cm^{-1}, 140 cm^{-1}, 117 cm^{-1} and 104 cm^{-1}). According to the previous report[1], the peaks of 1T′ phase $MoTe_2$ are obviously different from the trimerized structure of *d*1T-$MoTe_2$. Instead, the peaks of the measured Raman spectra can match well with our calculations on the sample with trimerized structure.

Supplementary Table 1. LDA calculated Gamma point Phonon frequency (cm^{-1}) and the relative irreducible representation for the *d*1T, 2H and 1T phase $MoTe_2$, the Raman (R) and Infrared (I) activity are also indicated. Due to the absentence of the inversion center, all the modes in *d*1T-$MoTe_2$ are both Raman and infrared active.

*d*1T $MoTe_2$			2H $MoTe_2$ 1L			1T $MoTe_2$		
Wavenumber	Mode	Optional activity	Wavenumber	Mode	Optional activity	Wavenumber	Mode	Optional activity
0	E	I+R	0	E’	I	0	A_{2u}	I
0	A_1	I+R	0	A’’$_2$	I	0	E_u	I
80.69	E	I+R	119.1	E’’	R	105.7	E_g	R
105.61	E	I+R	174.1	A’$_1$	R	157.9	A_{1g}	R
106.05	A_1	I+R	239.3	E’	I	165.3	E_u	I
111.39	A_2	I+R	296.1	A’’$_2$	R	244.1	A_{2u}	I
113.51	E	I+R						
132.43	A_2	I+R						
137.19	E	I+R						
141.15	A_1	I+R						
168.47	E	I+R						
172.97	A_1	I+R						
198.56	E	I+R						
224.63	E	I+R						
237.08	A_2	I+R						
240.2	A_1	I+R						
288.41	E	I+R						
302.25	A_1	I+R						

Supplementary Note 2. Phase transition of the $MoTe_2$ nanosheets

The $MoTe_2$ nanosheets were exfoliated onto single graphene layer. The optical microscopy (OM) image of $MoTe_2$ nanosheets is shown in Supplementary Figure 2a. Supplementary Figure 2b illustrates AFM image and corresponding AFM height curve of the $MoTe_2$, revealing the 5 layers nanosheet thickness of ～4 nm. Supplementary Figure 2c reveals cross-section TEM image of 5 layers $MoTe_2$ nanosheets. The intensity of the graphene is observed to be approximately twice as high for the 2D peak at 2700 cm^{-1} as for the G peak at 1580 cm^{-1}, as shown in Supplementary Figure 2d. The full-width half-maximum of 2D peak is less than 30 cm^{-1}. These results indicate that the graphene used is monolayer. The structural phases of 5 layers $MoTe_2$ nanosheets onto monolayer graphene were transformed by laser irradiation and Raman spectra of $MoTe_2$ nanosheets were measured, as shown in Supplementary Figure 2e,f. The pristine 2H phase $MoTe_2$ nanosheets exhibit three main Raman modes, inculding layer-sensitive B_{2g} mode near 290 cm^{-1}. The laser-irradiated nanosheets appear a new set of Raman peaks, demonstrating the non-centrosymmetric trimerized structure of *d*1T-$MoTe_2$.

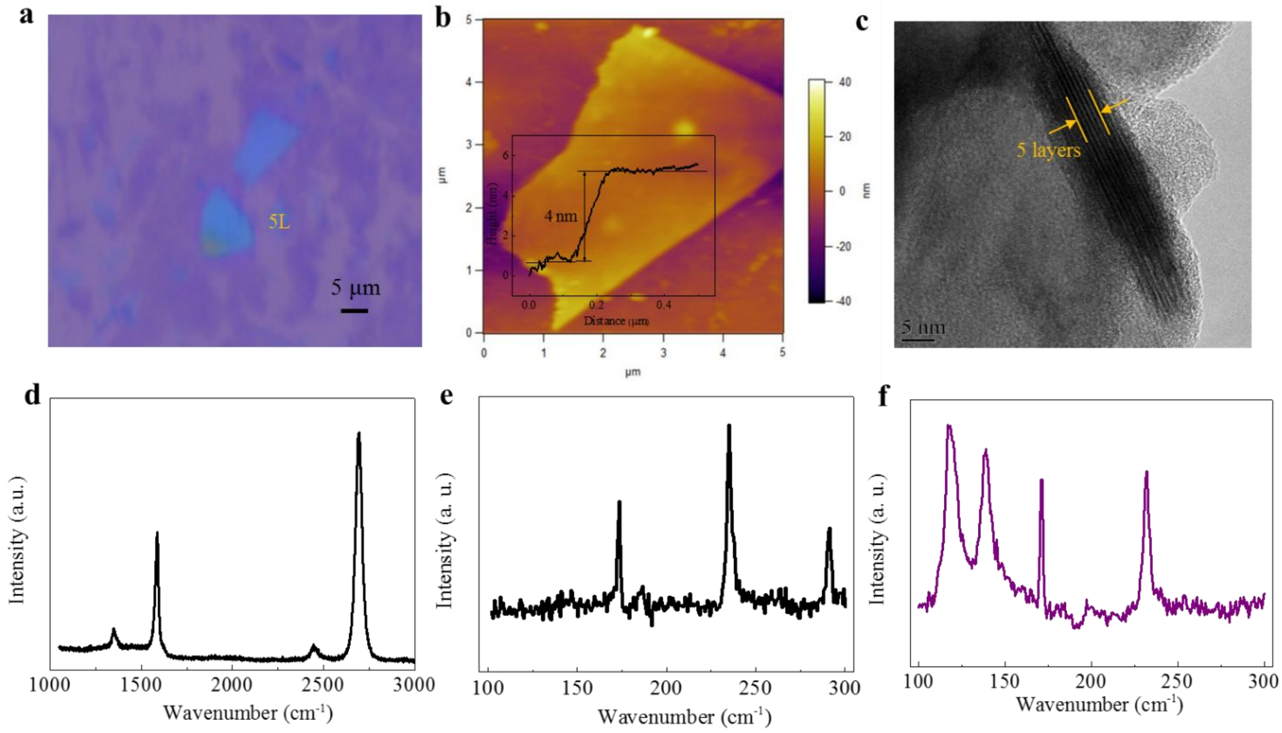

Supplementary Figure 2. Phase transition of the $MoTe_2$ nanosheets onto monolayer graphene. a, OM image of $MoTe_2$ nanosheets onto monolayer graphene. **b**, AFM image and height curve of 5 layers *d*1T-$MoTe_2$ nanosheets onto monolayer graphene. **c**, Cross-sectional TEM image of 5 layers *d*1T-$MoTe_2$ on monolayer

graphene. **d**, Raman spectrum of monolayer graphene layer used. **e**,**f**, Raman spectra of 5 layers 2H-$MoTe_2$ and *d*1T-$MoTe_2$ nanosheets onto monolayer graphene.

Supplementary Note 3. Ferroelectricity in $MoTe_2$ by PFM

Supplementary Figure 3a shows the out-of-plane PFM phase hysteretic loops of *d*1T-$MoTe_2$ nanosheets with different layer number made onto Pt substrate. The phase differnence of all curves is around 180°, indicating no obvious thickness limited ferroelectricity. Supplementary Figure 3b presents out-of-plane PFM phase hysteretic loop of 5 layers *d*1T-$MoTe_2$ nanosheets onto monolayer graphene. The phase difference between the two polarization states remains 180°, implying the existence of ferroelectricity in $MoTe_2$ samples even when fabricated on different substrates. In comparison, to rule out the possibility with non-ferroelectric materials, the phase hysteretic loops measured at random locations on the surface of the 2H and *d*1T phase monolayer $MoTe_2$, as shown in Supplementary Figure 3c,d. There is almost no phase contrast in pristine 2H-$MoTe_2$, indicating the ferroelectricity is absent in 2H phase[2], but the *d*1T-$MoTe_2$ shows the obvious ferroelectric hysteretic behaviour. Supplementary Figure 3e,f shows in-plane PFM amplitude and phase images of *d*1T-$MoTe_2$ nanosheets onto Pt substrate. No phase contrast was found in the samples, revealing that ferroelectric polarization is absent in the *d*1T-$MoTe_2$ along in-plane direction. Supplementary Table 2 gives a summary of the critical or thinnest thickness and T_c with previously reported ferroelectric materials by experiments. So far, monolayer *d*1T-$MoTe_2$ showing room-temperature out-of-plane ferroelectricty is thinnest scale among nanoscale ferroelectric materials which have been reported[3-12].

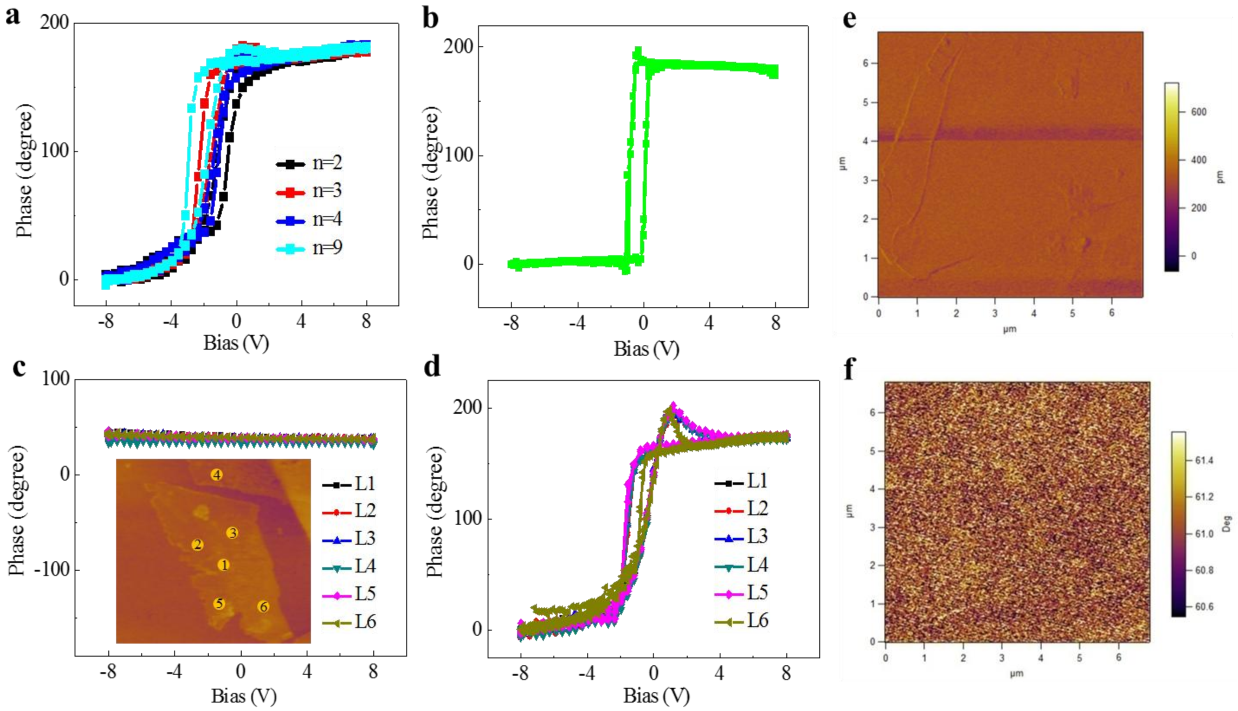

Supplementary Figure 3. Ferroelectricity in $MoTe_2$ by PFM. a, Phase signals of *d*1T-$MoTe_2$ nanosheets with different layer number onto Pt substrate (layer number n=2, 3, 4, 9). **b**, The PFM phase hysteretic loop of 5 layers *d*1T-$MoTe_2$ nanosheets onto monolayer graphene. **c**,**d**, PFM phase hysteretic loops of 2H and *d*1T-$MoTe_2$ taken at random locations, the inset shows the randomly selected locations. **e**,**f**, In-plane PFM amplitude (top) and phase images (bottom) of *d*1T-$MoTe_2$ with 5 layers.

Supplementary Table 2. Comparison of the critical or thinnest thickness and Curie temperature (T_c) with the previously reported common ferroelectric materials possessing out-of-plane ferroelectricity by experiments (RT represents room temperature).

Ferroelectric materials	Critical or thinnest thickness	Curie temperature (T_c)	Polarization direction	Reference
$BaTiO_3$	1 nm	Above RT	Out-of-plane	4
$PbTiO_3$	1.2 nm	～549 K	Out-of-plane	3
PVDF	～2.2 nm	Above RT	Out-of-plane	11
$BiFeO_3$	2 nm	Above RT	Out-of-plane	12
$CuInP_2S_6$	4 nm	～323 K	Out-of-plane	5
WTe_2	1.4 nm	～350 K	Out-of-plane	10
In_2Se_3	3 nm	Above RT	Out-of-plane	6-9
$MoTe_2$	0.8 nm	～338 K	Out-of-plane	This work

Supplementary Note 4. The $MoTe_2$ film by MBE

Raman spectra and surface topography were measured in the monolayer $MoTe_2$ film onto HOPG and SiC substrates, respectively. Supplementary Figure 4a shows Raman spectra of monolayer MBE grown $MoTe_2$ after laser processing. The laser-irradiated films appear Raman peaks, corresponding to *d*1T phase $MoTe_2$. AFM image of monolayer $MoTe_2$ film is shown in Supplementary Figure 4b. In addtion, atomic-scale STM image of monolayer $MoTe_2$ films is also presented in Supplementary Figure 4c. These images indicate the smooth morphology of the samples. Supplementary Figure 4d-g shows PFM phase and amplitude loops of monolayer *d*1T-$MoTe_2$ films onto HOPG and SiC substrates, respectively. Similar to the exfoliated samples described in the main text, the 180 ° phase changes provide a clear evidence of the switchable polarization. Supplementary Figure 4h shows SHG spectrum from monolayer *d*1T-$MoTe_2$. A femtosecond-pulsed laser generates SHG signals in reflection with wavelength of 990 nm. The SHG signal (λ = 495 nm) generated from monolayer *d*1T-$MoTe_2$, which originates from the non-centrosymmetric *d*1T-$MoTe_2$.

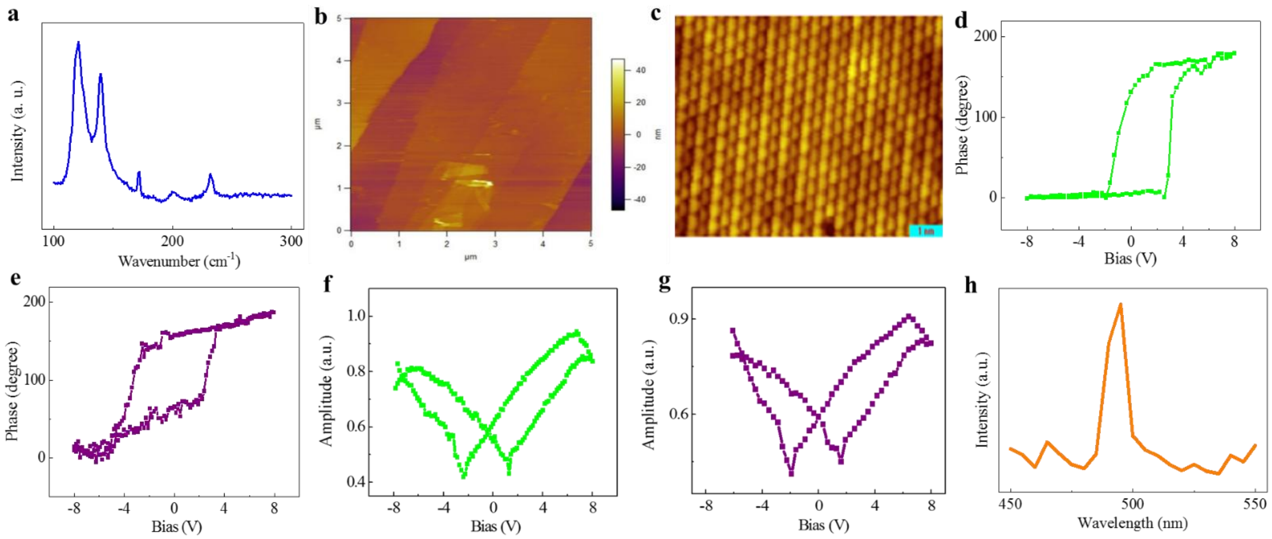

Supplementary Figure 4. MBE-grown $MoTe_2$ films and characterizations. a, Raman spectra of monolayer *d*1T-$MoTe_2$ film by MBE. **b**, Surface topography of monolayer $MoTe_2$ film by MBE. **c**, STM image of monolayer $MoTe_2$ films by MBE. **d**,**e**, PFM phase loops of monolayer $MoTe_2$ films deposited onto HOPG and SiC substrate. **f**,**g**, PFM amplitude loops of monolayer $MoTe_2$ films deposited onto HOPG and SiC substrate. **h**, Second harmonic generation spectrum from monolayer *d*1T-$MoTe_2$.

Supplementary Note 5. First-principles calculation

Monolayer 2D layered materials have been predicted to exhibit novel ferroelectric phenomena by using theoretical calculation[13-24]. Here, first-principles calculation was used to investigate the microscopic origin of the ferroelectricity in *d*1T-$MoTe_2$. Among the existing phases of MoX_2, the metastable 1T phase $MoTe_2$ is a high symmetry phase where the Mo atoms are coordinated to the center of the six Te ligands in an octahedral arrangement. As a result, the Mo site is also the inversion center of the centrosymmetric 1T phase, as shown in the inset of Supplementary Figure 5a. The spontaneous symmetry breaking of the 1T-$MoTe_2$ gives the possibility of generating the ferroelectricity in monolayer $MoTe_2$. For this purpose, we first analyzed the structural stability of the 1T-$MoTe_2$ through its phonon dispersion calculated within the density functional perturbation theory. From the phonon dispersion of the high symmetry paraelectric 1T-$MoTe_2$, there are many imaginary phonon frequencies indicating the instability of the structure. Since there are some soft modes from the phonon dispersion of 1T-$MoTe_2$, suggesting the possible phase transition in this system. By freezing the eigen-displacement of the unstable modeat M point, the 1T′-$MoTe_2$ structure is obtained with the characteristic Mo-Mo zigzag chain. However, the inversion symmetry remains in the 1T′-$MoTe_2$, excluding its possibility as a ferroelectric candidate. The structural distortion associated with the K point instability leads to the trimerized $\sqrt{3}\times\sqrt{3}$ *d*1T-$MoTe_2$ structure, which is a stable phase from thermodynamics as no soft mode exists in its calculated phonon dispersion. The energy barrier between *d*1T and 1T-$MoTe_2$ is shown in Supplementary Figure 5b. The *d*1T phase is more stable than 1T phase. The *d*1T structure could be constructed from two steps, only the adjacent three Mo atoms move laterally to form the equilateral triangle (we refer it as the trimerized structure) and then the ions atoms move in the z direction to form the final *d*1T structure. Our in-depth DFT calculations suggest that the spontaneous polarization is mainly from the process of the trimerization with the polarization coming from the electronic part, while the Te atoms' movements in z direction due to the depolarization filed slightly weaken the polarization. As a result, the *d*1T structure is 0.47 eV lower than the trimerized structure. The physical picture is quite different from the traditional improper ferroelectric such as hexagonal manganite $YMnO_3$, where the trimerization has negligible polarization and the major contribution of the polarization is from the distortion caused by the out of plane ionic displacement (Γ_2^- polar mode)[25]. According to the changes of the dipole moment and taken the effectively thickness of the

monolayer $MoTe_2$ as 5 Å, a spontaneous polarization of 0.68 $\mu C/cm^2$ is obtained for the *d*1T structure (Supplementary Figure 5c-f).

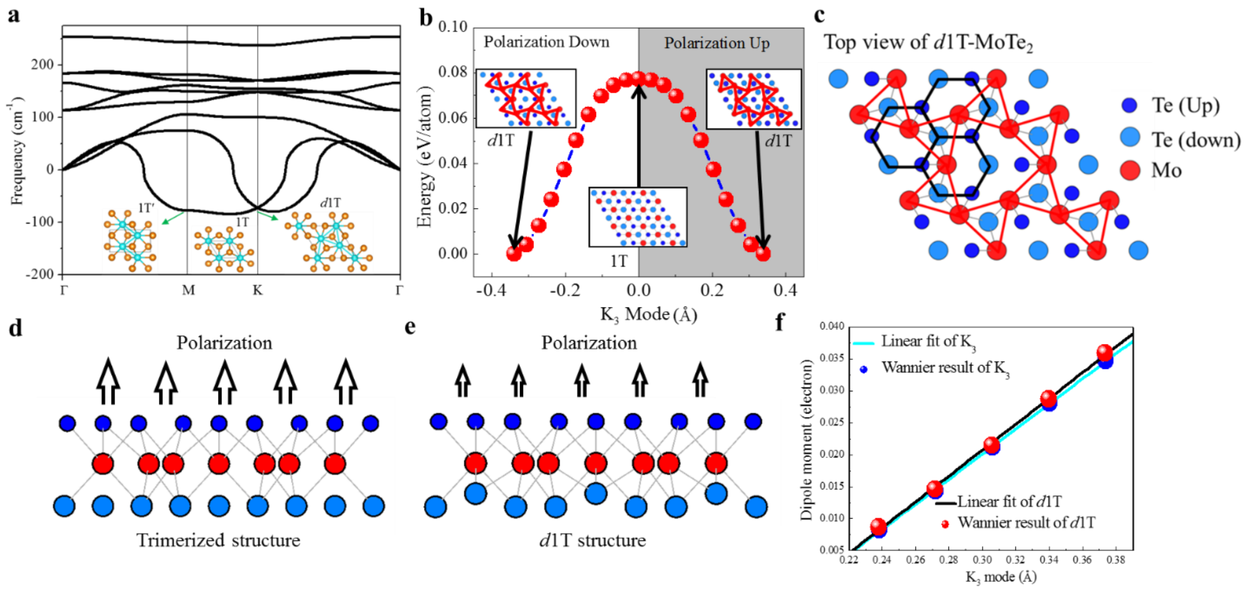

Supplementary Figure 5. First-principles calculation. a, Phonon dispersion of the 1T-$MoTe_2$, with the structure of 1T shown in the middle. The unstable modes in M and K points lead to the 1T′ and *d*1T-$MoTe_2$, respectively. **b**, Energy barrier between *d*1T and 1T-$MoTe_2$ (Lable the 1T below the image at the lower middle, and the *d*1T at the upper image). **c**, The top view of the trimerized/*d*1T-$MoTe_2$, the structures show no difference in trimerized and *d*1T-$MoTe_2$ in the top view. The side view shows the difference between the (**d**) trimerized and (**e**) *d*1T structure. **f**, The change of the dipole moment in the *d*1T phase and the trimerized structure.

Supplementary Note 6. Temperature dependent PFM measurement

Supplementary Figure 6a shows temperature dependence of PFM phase loops of 5 layers *d*1T-$MoTe_2$ nanosheets. A transition temperature of 5 layers *d*1T-$MoTe_2$ is about 318 K. The hysteretic phenomena disappears when the temperature is over 318 K. Supplementary Figure 6b shows PFM phase loops of 16 layers *d*1T-$MoTe_2$ nanosheets at room temperature. The AFM image and height profile of 16 layers $MoTe_2$ nanosheets were presented (Supplementary Figure 6c,d). There is no obvious hysteretic loop in 16 layers *d*1T-$MoTe_2$, indicating lack of room-temperature ferroelectricity in this case.

In the *d*1T-$MoTe_2$, it is found that with increasing layer thickness, the energy difference between the ferroelectric *d*1T phase and paraelectric 1T phase is decreasing, as shown in

Supplementary Figure 7. Since the ferroelectric transition temperature is proportional to the energy barrier between the 1T and *d*1T phase, this result suggests the decreasing transition temperature is an intrinsic effect of the d1T-$MoTe_2$, which may not related with the extrinsic effect such as the Te vacancies. The trimerization can have larger energy gain in thinner thickness compared with the bulk, therefore the quantum confinement should play a critical role in this anomalous phase transition phenomenon, and the details require further studies.

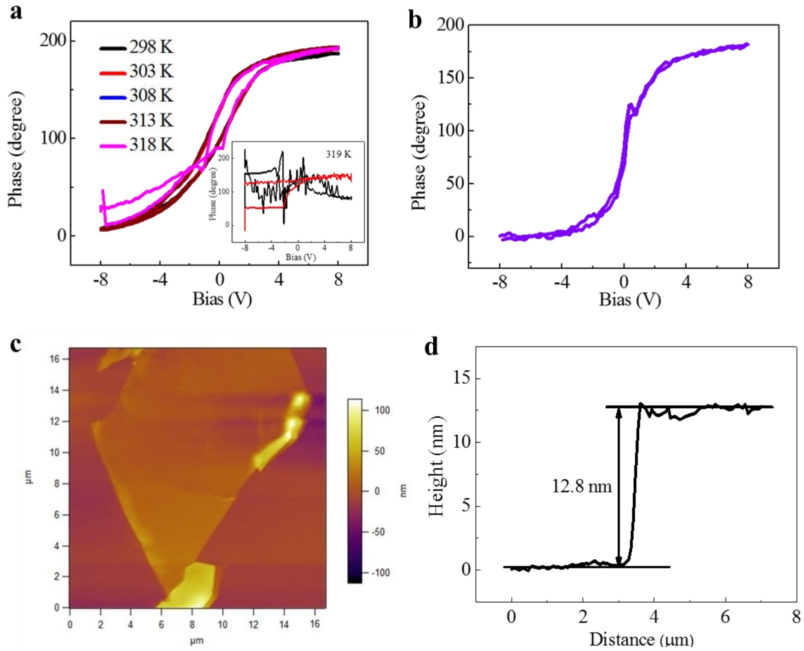

Supplementary Figure 6. Temperature dependence of PFM measurement. a, Temperature dependence of PFM phase loops of 5 layers *d*1T-$MoTe_2$ nanosheets. **b**, PFM phase loops of 16 layers *d*1T-$MoTe_2$ nanosheets at room temperature. **c**,**d**, AFM image and height profile of $MoTe_2$ nanosheets with 16 layers.

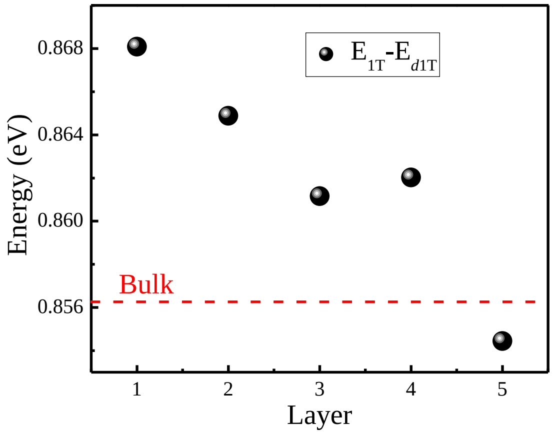

Supplementary Figure 7. Energy difference of the *d*1T phase and 1T phase with increasing layer thicknesses.

Supplementary Note 7. The bandstructure of the van der Waals heterostructure

As suggested in our calculations, the bandstructure of the van der Waals (vdW) heterostructure is similar to the superimposition of the bandstructure of individual *d*1T-$MoTe_2$ and graphene, as shown in Supplementary Figure 8. The vdW interaction has little effect on the intrinsic physical properties of the *d*1T-$MoTe_2$. Especially, for the 2D vdW ferroelectric tunneling junction, the physical properties of the component layers are largely unaffected due to the weak vdW interaction, allowing for the design of heterojunction devices with desired functionality.

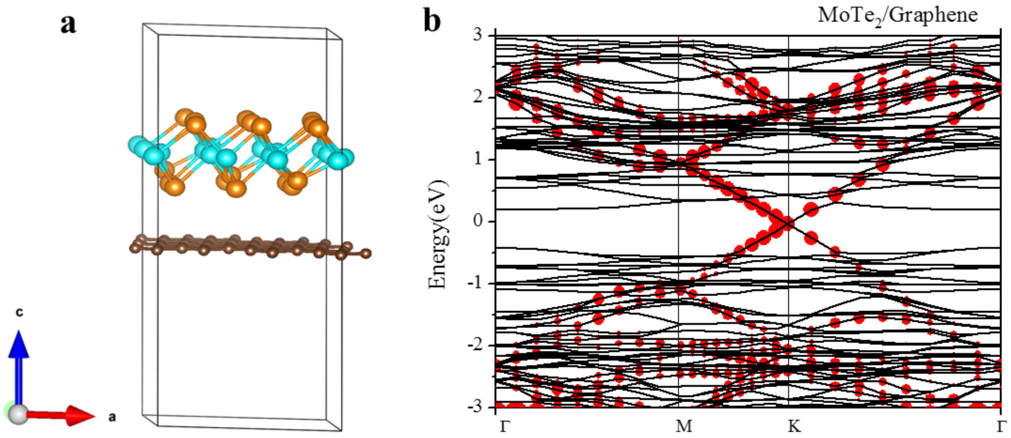

Supplementary Figure 8. The bandstructure of the vdW heterostructure. a, Structure illustration of the *d*1T-$MoTe_2$ and graphene heterostructure, as well as the band structure of the heterostructures. **b**, The characteristic band of the graphene is highlighted with the red ball, with the size proportional to the weights of the electronic states on to the C atom.

Supplementary References

1. Wang, J. et al. Determination of crystal axes in semimetallic T'-$MoTe_2$ by polarized Raman spectroscopy. *Adv. Funct. Mater.* **27**, 1604799 (2017).
2. Owczarek, M. et al. Flexible ferroelectric organic crystals. *Nat. Commun.* **7**, 13108 (2016).
3. Fong, D. D. et al. Ferroelectricity in ultrathin perovskite films. *Science* **304**, 1650 (2004).
4. Garcia, V. et al. Giant tunnel electroresistance for non-destructive readout of ferroelectric states. *Nature* **460**, 81 (2009).
5. Liu, F. et al. Room-temperature ferroelectricity in $CuInP_2S_6$ ultrathin flakes. *Nat. Commun.* **7**, 12357 (2016).
6. Zhou, Y. et al. Out-of-Plane piezoelectricity and ferroelectricity in layered α-In_2Se_3

nanoflakes. *Nano Lett.* **17**, 5508 (2017).

7. Cui, C. et al. Intercorrelated in-plane and out-of-plane ferroelectricity in ultrathin two-dimensional layered semiconductor In_2Se_3. *Nano Lett.* **18**, 1253 (2018).
8. Xiao, J. et al. Intrinsic two-dimensional ferroelectricity with dipole locking. *Phys. Rev. Lett.* **120**, 227601 (2018).
9. Poh, S. M. et al. Molecular-beam epitaxy of two-dimensional In_2Se_3 and its giant electroresistance switching in ferroresistive memory junction. *Nano Lett.* **18**, 6340 (2018).
10. Fei, Z. et al. Ferroelectric switching of a two-dimensional metal. *Nature* **560**, 336 (2018).
11. Tian, B. B. et al. Tunnel electroresistance through organic ferroelectrics. *Nat. Commun.* **7**, 11502 (2016).
12. Béa, H. et al. Ferroelectricity down to at least 2 nm in multiferroic $BiFeO_3$ epitaxial thin films. *Jpn. J. Appl. Phys.* **45**, L187 (2006).
13. Li, W. & Li, J. Ferroelasticity and domain physics in two-dimensional transition metal dichalcogenide monolayers. *Nat. Commun.* 7, 10843 (2016).
14. Seixas, L., Rodin, A. S., Carvalho, A. & Castro Neto, A. H. Multiferroic two-dimensional materials. *Phys. Rev. Lett.* **116**, 206803 (2016).
15. Li, L. & Wu, M. Binary compound bilayer and multilayer with vertical polarizations: two-dimensional ferroelectrics, multiferroics, and nanogenerators. *ACS Nano* **11**, 6382 (2017).
16. Wu, M. & Zeng, X. C. Intrinsic ferroelasticity and/or multiferroicity in two-dimensional phosphorene and phosphorene analogues. *Nano Lett.* **16**, 3236 (2016).
17. Wu, M. et al. Ferroelectricity in covalently functionalized two-dimensional materials: integration of high-mobility semiconductors and nonvolatile memory. *Nano Lett.* **16**, 7309 (2016).
18. Xiao, C. et al. Elemental ferroelectricity and antiferroelectricity in group-V monolayer. *Adv. Funct. Mater.* **28**, 1707383 (2018).
19. Xu, B. et al. Monolayer $AgBiP_2Se_6$: an atomically thin ferroelectric semiconductor with out-plane polarization. *Nanoscale* **9**, 8427 (2017).
20. Wan, W., Liu, C., Xiao, W. & Yao, Y. Promising ferroelectricity in 2D group IV tellurides: a first-principles study. *Appl. Phys. Lett.* **111**, 132904 (2017).
21. Wang, H. & Qian, X. Giant optical second harmonic generation in two-dimensional

multiferroics. *Nano Lett.* **17**, 5027 (2017).

22. Yang, Q. et al. Chemically functionalized phosphorene: two-dimensional multiferroics with vertical polarization and mobile magnetism. *J. Am. Chem. Soc.* **139**, 11506 (2017).
23. Wu, M. & Zeng, X. C. Bismuth oxychalcogenides: a new class of ferroelectric/ ferroelastic materials with ultra high mobility. *Nano Lett.* **17**, 6309 (2017).
24. Hu, T. et al. New ferroelectric phase in atomic-thick phosphorene nanoribbons: existence of in-plane electric polarization. *Nano Lett.* **16**, 8015 (2016).
25. Fennie, C. J. & Rabe, K. M. Ferroelectric transition in $YMnO_3$ from first principles. *Phys. Rev. B* **72**, 100103(R) (2015).
